# Letter to the Editor Re: Scott S.N., et al. Nutrients 2019, 11(5), 1022

**DOI:** 10.3390/nu11112700

**Published:** 2019-11-07

**Authors:** Jake A. Kushner

**Affiliations:** McNair Interests, 109 N. Post Oak Lane, Suite 600, Houston, 77024 TX, USA; jake.kushner@mac.com; Tel.: +1-(713)-336-7888

I read with great interest the thoughtful review concerning the potential impact of carbohydrate restriction in type 1 diabetes on glycaemic parameters and athletic performance [1]. In their review, the authors incorrectly assert that the 2018 American Diabetes Association (ADA) Standards of Medical Care in Diabetes endorses a specific dietary distribution of calories among carbohydrates, fats, and proteins for people with diabetes: ***“The ADA Standards of Medical Care in Diabetes (2018) recommend 15–20% of total energy from protein, 20–35% of energy from dietary fat, which leaves a balance of 45–60% energy from carbohydrate* [25].”**

Although several passages within the 2018 ADA Standards relate to macronutrient distribution, none prescribe a discrete proportion of dietary fat, protein, or fat [2]. Indeed, regarding “Eating patterns and macronutrient distribution”, the ADA Standards of Care explicitly advises against a specific macronutrient distribution: ***“There is no single ideal dietary distribution of calories among carbohydrates, fats, and proteins for people with diabetes; therefore, macronutrient distribution should be individualized while keeping total calorie and metabolic goals in mind.”***

Similarly, in regards to protein intake, the 2018 ADA Standards unambiguously advises in favor of individualization: “There is no evidence that adjusting the daily level of protein intake (typically 1–1.5 g/kg body weight/day or 15–20% total calories) will improve health in individuals without diabetic kidney disease, and research is inconclusive regarding the ideal amount of dietary protein to optimize either glycemic control or cardiovascular disease (CVD) risk (72). Therefore, protein intake goals should be individualized based on current eating patterns.”

Finally, with regards to dietary fat, the 2018 ADA Standards emphasizes avoiding saturated fats but does not limit total fat. The 2018 ADA Standards briefly mentions that the topic is controversial, includes reference to (without endorsing) the National Academy of Medicine macronutrient distributions: ***“The ideal amount of dietary fat for individuals with diabetes is controversial. The National Academy of Medicine has defined an acceptable macronutrient distribution for total fat for all adults to be 20–35% of total calorie intake (92). The type of fats consumed is more important than total amount of fat when looking at metabolic goals and CVD risk, and it is recommended that the percentage of total calories from saturated fats should be limited (93–97).”***

People with diabetes may benefit from dietary options to maximize health and wellness. Thankfully, the ADA Standards of Medical Care allow for individualization of macronutrient distribution.
